# Risk of Hypoglycemia and Associated Factors Among In‐Hospital Chinese Patients With Latent Autoimmune Diabetes in Adults (LADA): A Multicenter Retrospective Cohort Study

**DOI:** 10.1155/jdr/2961800

**Published:** 2026-06-29

**Authors:** Haojie Zhou, Qiang Zhang, Guniqing Zhu, Weiwei Xu, Xueyan Zhao, Xiangfu Gu, Xiaoyu Cai, Xiaoli Zhu, Ran Li

**Affiliations:** ^1^ Department of Cardiovascular Medicine, The Second Affiliated Hospital of Chongqing Medical University, Chongqing, China, cqmu.edu.cn; ^2^ School of Nursing, Dali University, Dali, China, dali.edu.cn; ^3^ The First School of Clinical Medicine, Southern Medical University, Guangzhou, China, fimmu.com; ^4^ Department of Endocrinology and Metabolism, The Second Affiliated Hospital of Chongqing Medical University, Chongqing, China, cqmu.edu.cn; ^5^ Department of Endocrinology, The First Affiliated Hospital of Dali University, Dali, China, dali.edu.cn; ^6^ Patient Service Center, The First Affiliated Hospital of Dali University, Dali, China, dali.edu.cn; ^7^ Department of Critical Care Medicine, The Second Affiliated Hospital of Chongqing Medical University, Chongqing, China, cqmu.edu.cn

**Keywords:** associated factors, hypoglycemia, latent autoimmune diabetes in adults (LADA)

## Abstract

**Background:**

Latent autoimmune diabetes in adults (LADA) is characterized by progressive *β*‐cell dysfunction and glycemic lability, which increases susceptibility to in‐hospital hypoglycemia. Real‐world data on the epidemiology of hypoglycemia and associated factors among LADA inpatients are limited, particularly among multiethnic populations in Southwest China. This study is aimed at exploring the prevalence, regional heterogeneity, and independent associated factors of in‐hospital hypoglycemia in the LADA population.

**Methods:**

Following the STROBE guidelines, 709 hospitalized patients with LADA from five tertiary hospitals in Southwest China from January 2019 to September 2025 were enrolled retrospectively. Missing data were imputed via multiple imputation by chained equations with results pooled using Rubin′s rules. Univariate logistic regression was applied for preliminary factor screening. Multicollinearity was evaluated prior to multivariate regression. Model performance was assessed using the AUC for discriminative ability and Nagelkerke′s *R* ^2^ for goodness of fit, with subset sensitivity analysis performed to verify result robustness.

**Results:**

The overall in‐hospital hypoglycemia incidence was 47.39%, with evident regional heterogeneity across Southwest China. After pooling results from 20 imputed datasets, seven independently associated factors were identified. These factors included insulin pump therapy (OR = 8.233, 95% CI: 3.914–17.274), the largest amplitude of glycemic excursions (OR = 1.136, 95% CI: 1.081–1.193), length of hospital stay (OR = 1.134, 95% CI: 1.074–1.197), prior hypoglycemia history (OR = 2.447, 95% CI: 1.448–4.024), sex (OR = 1.655, 95% CI: 1.065–2.574), geographic origin (OR = 0.369, 95% CI: 0.225–0.605), and glycated hemoglobin (OR = 0.902, 95% CI: 0.837–0.973). The median AUC and Nagelkerke′s *R* ^2^ demonstrated satisfactory discrimination and calibration of the final model.

**Conclusions:**

Hospitalized LADA patients in Southwest China have a high prevalence of in‐hospital hypoglycemia with prominent regional disparities. The identified associated factors support individualized risk stratification, targeted glycemic monitoring, and optimized inpatient management, helping reduce hypoglycemia burden and improve glycemic safety among hospitalized LADA patients.

## 1. Introduction

Latent autoimmune diabetes in adults (LADA) is a unique, slowly progressive subtype of autoimmune diabetes with intermediate clinical and pathophysiological characteristics between classic Type 1 diabetes mellitus (T1DM) and Type 2 diabetes mellitus (T2DM) [1]. Characterized by insidious adult onset and gradual, irreversible pancreatic *β*‐cell dysfunction, LADA inherits the autoimmune pathogenesis of T1DM while exhibiting the metabolic heterogeneity of T2DM, forming a distinct diabetes phenotype with variable disease progression and prominent glycemic lability [2]. Epidemiological studies have demonstrated that LADA accounts for 2%–10% of all adult‐onset diabetes cases worldwide, with substantial disparities across ethnic populations, geographical regions, and autoantibody screening strategies, indicating notable population‐based heterogeneity in disease presentation and prognosis [3]. Unlike conventional T1DM and T2DM, the atypical and progressive disease course of LADA frequently results in delayed diagnosis and suboptimal glycemic management, rendering this subtype a major challenge for routine diabetes care [4].

Precision and individualized glycemic management remain the cornerstone of modern diabetes treatment, aimed at maintaining stable glycemic status and prevent acute and chronic diabetic complications [5]. Optimized glycemic control effectively delays the progression of microvascular and macrovascular lesions and significantly improves long‐term prognostic outcomes in diabetic patients [6, 7].

As a common, therapy‐associated acute complication with life‐threatening potential, hypoglycemia substantially impairs the safety of antihyperglycemic therapy and patient quality of life, and is closely associated with increased hospitalization burden and mortality risks [8]. Previous studies have documented a high hypoglycemia prevalence of 48.8% among Chinese patients with T1DM [9]. However, the true hypoglycemia burden in the LADA subtype remains considerably underestimated due to the lack of targeted epidemiological data and subtype‐specific monitoring criteria [10]. The progressive deterioration of residual *β*‐cell function and inherent glycemic instability render LADA patients more susceptible to glycemic excursions and hypoglycemic events [11], yet high‐quality clinical evidence focusing on hypoglycemia susceptibility in LADA populations remains limited.

Individual hypoglycemia risk is modulated by a combination of modifiable and nonmodifiable factors, including residual pancreatic *β*‐cell function, insulin sensitivity, dietary patterns, and systemic metabolic status [12]. Hospitalized diabetic patients represent a particularly high‐risk population for hypoglycemia, as acute in‐hospital stress, frequent therapeutic adjustments, and complicated comorbidities collectively disrupt glycemic homeostasis and substantially impair inpatient glycemic safety [13]. Despite remarkable advances in glucose monitoring techniques and antidiabetic pharmacotherapy in recent years, balancing intensive glycemic control with effective hypoglycemia prevention remains a key unmet challenge in inpatient diabetes management [14].

Current clinical evidence regarding the epidemiology of hypoglycemia and its associated factors is largely based on patients with T1DM and T2DM [15]. In contrast, real‐world data focusing on hypoglycemia profiles in hospitalized LADA patients remain scarce globally. Most conventional LADA management strategies rely on empirical insulin‐based regimens [16]. Although emerging antidiabetic agents, including GLP‐1 receptor agonists and novel dual agonists, have demonstrated favorable efficacy in stabilizing glycemic fluctuations and reducing hypoglycemia risk among ambulatory LADA patients in existing trials [17, 18], relevant clinical evidence for LADA inpatient management remains insufficient. Notably, Southwest China is characterized by multiethnic compositions, distinct dietary structures, and regional lifestyle characteristics, which may shape region‐specific glycemic phenotypes and hypoglycemia risk patterns. Nevertheless, real‐world inpatient evidence regarding hypoglycemia characteristics and tailored management strategies for LADA in this region remains inadequate, creating an unaddressed subtype‐specific and regional research gap.

Against this background, the present multicenter retrospective study is aimed at exploring the prevalence, regional heterogeneity, and independent associated factors of in‐hospital hypoglycemia among LADA patients in Southwest China. The findings are expected to provide targeted real‐world evidence for individualized glycemic risk stratification and optimized hypoglycemia prevention strategies, thereby improving inpatient glycemic safety and clinical outcomes for LADA patients.

## 2. Materials and Methods

### 2.1. Study Participants

This retrospective cohort study was conducted following the Strengthening the Reporting of Observational Studies in Epidemiology (STROBE) guidelines [19]. Participants were consecutively enrolled from five tertiary hospitals across Southwest China between January 2019 and September 2025.

Inclusion criteria were as follows: (1) diagnosed with LADA according to the 2021 Chinese Expert Consensus on the Diagnosis and Treatment of Latent Autoimmune Diabetes in Adults [20]. The core serological markers for LADA diagnosis included glutamic acid decarboxylase antibody, insulinoma‐associated antigen‐2 antibody, zinc transporter 8 antibody, islet cell antibody, and insulin autoantibody. All antibodies were measured using chemiluminescence immunoassay. Positive cut‐off values were uniformly applied across all participating centers in accordance with the manufacturer′s instructions; (2) age at diabetes onset ≥ 18 years; and (3) no insulin dependence within the first 6 months after initial diagnosis. Insulin independence was defined as sustained glycemic control achieved via lifestyle intervention or oral hypoglycemic agents, with no regular administration of exogenous insulin during this period [16].

Exclusion criteria were the (1) presence of other concurrent autoimmune diseases and (2) missing core clinical or laboratory data.

This study was approved by the Ethics Committees of the First Affiliated Hospital of Dali University (No. DFY20250520002) and the Ethics Committee of the Second Affiliated Hospital of Chongqing Medical University (No. 275 [2025]). As the leading ethical review bodies for this multicenter study, their approvals were fully recognized by the other three participating hospitals, and additional ethical review was therefore waived. All procedures were performed in compliance with the principles of the Declaration of Helsinki.

### 2.2. Research Methods

A standardized data collection checklist was formulated with expert consultation based on the Delphi method to unify variable classification, covering three dimensions: (1) sociodemographic data, including age, gender, marital status, and ethnicity; (2) clinical characteristics, encompassing medical history, diabetic complications, and insulin administration; (3) laboratory parameters, involving routine blood tests, glycated hemoglobin (HbA1c), and hepatic and renal function. All raw clinical and laboratory data were retrospectively extracted from the electronic medical record (EMR) systems of participating hospitals in accordance with the unified checklist. All variables were measured prior to the onset of the first in‐hospital hypoglycemic episode. For patients without hypoglycemia during hospitalization, the last set of data recorded before discharge was adopted.

The primary endpoint was the first episode of capillary blood glucose below 3.9 mmol/L during hospitalization [21], which was defined as a binary outcome (presence or absence of hypoglycemia). Hypoglycemic events were comprehensively ascertained through daily blood glucose monitoring records, physician progress notes, and nursing logs.

### 2.3. Missing Data Handling

All raw data were double‐entered independently using Microsoft Excel and cross‐checked to reduce manual entry errors. Any discrepancies were resolved by reviewing the original EMRs to ensure data accuracy. Missing data mainly resulted from incomplete medical documentation and unconducted laboratory tests. Since missing values were only associated with observed baseline covariates rather than unmeasured true values, the data satisfied the Missing at Random (MAR) assumption [22].

Variables with a missing rate > 30*%* were excluded due to insufficient valid cases and potential analytical bias. For variables with a missing rate ≤ 30*%*, multiple imputation by chained equations (MICE) was applied to minimize bias from complete‐case analysis [23].

All imputation procedures were implemented via the IterativeImputer module in scikit‐learn (Python 3.14.5). A fixed random seed was set to ensure result reproducibility, and 20 complete imputed datasets were generated. A random forest regression model containing 100 decision trees was adopted for imputation prediction, with a maximum of 20 iterations. Posterior sampling (sample posterior = true) was enabled to quantify imputation uncertainty. All covariates incorporated into the final statistical models were included in the imputation process to maximize information utilization. Separate analyses were conducted on each dataset, and pooled results were synthesized using Rubin′s rules [24].

### 2.4. Statistical Methods

All statistical tests were two‐tailed, and the significance level was set at *α* = 0.05.

#### 2.4.1. Data Description

This study had a relatively large sample size. Accordingly, the distribution of continuous variables was evaluated using skewness coefficients based on the central limit theorem. Variables with an absolute skewness value < 1 were defined as approximately normally distributed and presented as mean ± standard deviation. Nonnormally distributed variables were reported as median (interquartile range). Categorical variables were summarized as count (percentage).

#### 2.4.2. Between‐Group Comparisons

All participants were divided into hypoglycemia and nonhypoglycemia groups based on the occurrence of in‐hospital hypoglycemic events. Welch′s corrected independent *t*‐test was applied for approximately normal continuous variables. Mean differences and standard errors were calculated for each imputed dataset. Final pooled estimates of mean difference, standard error, and *p* value were synthesized using Rubin′s rules. For nonnormal continuous variables, rank transformation was performed in each dataset. Transformed ranks were treated as approximately normal data and analyzed using Welch′s *t*‐test. All group comparison results were finally pooled in accordance with Rubin′s rules.

Binary logistic regression was used for intergroup comparison of categorical outcomes. Grouping status was set as the independent variable. Corresponding odds ratios (OR) and 95% confidence intervals (95% CI) were calculated for each dataset. All regression outputs were pooled to obtain final pooled effect estimates.

#### 2.4.3. Univariate Logistic Regression Analysis

Univariate logistic regression was performed with in‐hospital hypoglycemia defined as the binary dependent outcome. Regression coefficients and standard errors were estimated separately for each of the 20 imputed datasets. Pooled OR, 95% CI, and *p* values were synthesized following Rubin′s rules. Variables with *p* < 0.1 in univariate analysis were included in subsequent collinearity assessment and multivariate modeling.

#### 2.4.4. Multicollinearity Assessment

All candidate variables screened by univariate analysis underwent multicollinearity evaluation. The variance inflation factor (VIF) was calculated for each imputed dataset. The average VIF across all datasets was used for judgment. A mean VIF greater than 10 indicates substantial multicollinearity [25].

Pairwise correlations were further analyzed to supplement VIF evaluation. Pearson correlation was used for continuous variable pairs. Point‐biserial correlation was adopted for continuous‐binary pairs. Phi correlation was applied for binary variable pairs. Average correlation coefficients across all datasets were calculated and visualized in a heatmap. The heatmap results provided supplementary evidence for multicollinearity judgment. Variables with severe multicollinearity were excluded before multivariate regression.

#### 2.4.5. Multivariate Logistic Regression Analysis

Variables that passed collinearity screening were simultaneously entered into the multivariate binary logistic regression model. Regression coefficients and covariance matrices were generated for each imputed dataset. Final pooled OR, 95% CI, and *p* values were calculated based on Rubin′s rules.

#### 2.4.6. Model Performance Evaluation and Sensitivity Analysis

Receiver operating characteristic (ROC) curves were plotted for each imputed dataset. The AUC value, optimal cutoff value, sensitivity, and specificity were calculated for each model. Model performance indices were summarized by median and range across all datasets. Nagelkerke′s *R* ^2^ was calculated to evaluate model goodness of fit and was descriptively summarized.

A preplanned sensitivity analysis was performed to verify result robustness regarding missing data processing. Ten imputed datasets were randomly selected for repeated multivariate modeling. Pooled effect estimates from the 10‐dataset subset were compared with results from the full 20‐dataset sample. Minor shifts in point estimates, overlapping 95% CIs, and consistent statistical significance between the two analyses confirmed the robustness of the primary analytic results.

## 3. Results

### 3.1. Incidence of In‐Hospital Hypoglycemia in LADA Patients

Prior to statistical analysis, systematic data quality control and missing value processing were performed for the original dataset. Variables with a missing rate exceeding 30% were excluded. MICE were applied to handle remaining missing data, and 20 complete imputed datasets were generated for subsequent analyses. The detailed missing value distribution of all variables is presented in Table S1.

**Figure 1:**
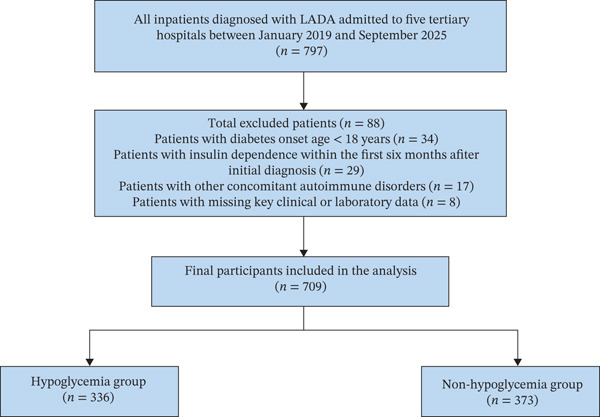
CONSORT‐style flow diagram of participant selection.

A total of 709 participants were enrolled in this study. The screening, inclusion, and exclusion procedures are illustrated in the CONSORT‐style flow diagram (Figure 1). The cohort consisted of 429 males (60.51%) and 280 females (39.49%). Participants′ age ranged from 18 to 84 years, with a median of 50 (39, 60) years. Based on the occurrence of in‐hospital hypoglycemia, all participants were divided into the hypoglycemia group (*n* = 336, 47.39%) and the nonhypoglycemia group (*n* = 373, 52.61%).

### 3.2. Baseline Characteristics and Intergroup Comparison

Pooled core baseline data are summarized in Table 1, and full intergroup comparison results of all measured variables are provided in Table S2.

**Table 1:** Core baseline characteristics of participants stratified by in‐hospital hypoglycemia status.

Variables		Total (*n* = 709)	Hypoglycemia group (*n* = 336)	Nonhypoglycemia group (*n* = 373)	*p* value
Age $\left(\bar{x}\pm s,\text{year}\right)$		50.24 ± 14.28	50.92 ± 13.60	49.62 ± 14.87	0.241
Sex (*n*, %)	Male	429 (60.50%)	186 (55.40%)	243 (65.10%)	0.010
	Female	280 (39.50%)	150 (44.60%)	130 (34.90%)	
Marital status (*n*, %)	Unmarried	59 (8.30%)	23 (6.80%)	36 (9.70%)	0.335
	Married	589 (83.10%)	279 (83.00%)	310 (83.10%)	
	Divorced	47 (6.60%)	26 (7.70%)	21 (5.60%)	
	Widowed	14 (2.00%)	8 (2.40%)	6 (1.60%)	
Ethnicity (*n*, %)	Han ethnicity	512 (72.20%)	224 (66.70%)	288 (77.20%)	0.002
	Ethnic minorities	197 (27.80%)	112 (33.30%)	85 (22.80%)	
Educational level (*n*, %)	Below senior high school	444 (62.60%)	228 (67.90%)	216 (57.90%)	0.008
	Senior high school and above	265 (37.40%)	108 (32.10%)	157 (42.10%)	
Geographic origin (*n*, %)	Yunnan Province	450 (63.50%)	254 (75.60%)	196 (52.50%)	< 0.001
	Chongqing Municipality	259 (36.50%)	82 (24.40%)	177 (47.50%)	
Body mass index (M [P_25_, P_75_], kg/m^2^)		21.38 (19.43, 23.48)	20.82 (18.93, 22.89)	21.72 (19.95, 23.91)	< 0.001
Length of hospital stay (M [P_25_, P_75_], day)		9.00 (7.00, 12.00)	10.00 (8.00, 13.00)	8.00 (7.00, 10.00)	< 0.001
Diabetes duration (M [P_25_, P_75_], year)		4.00 (0, 9.00)	6.00 (1.00, 10.00)	2.00 (0, 7.00)	< 0.001
Smoking history (*n*, %)	Yes	338 (47.40%)	154 (45.80%)	184 (49.30%)	0.392
	No	371 (52.30%)	182 (54.20%)	189 (50.70%)	
Prior hypoglycemia history (*n*, %)	Yes	136 (19.20%)	94 (28.00%)	42 (11.30%)	< 0.001
	No	573 (80.80%)	242 (72.00%)	331 (88.70%)	
Insulin pump therapy (*n*, %)	Yes	74 (10.40%)	61 (18.20%)	13 (3.50%)	< 0.001
	No	635 (89.60%)	275 (81.80%)	360 (96.50%)	
Largest amplitude of glycemic excursions ($\bar{x}\pm s$, mmol/L)		7.96 ± 4.38	9.29 ± 4.81	6.77 ± 3.56	< 0.001
Glycated hemoglobin ($\bar{x}\pm s$, %)		10.94 ± 3.11	10.73 ± 2.97	11.14 ± 3.22	0.090
Fasting C‐peptide (M [P_25_, P_75_], ng/mL)		0.37 (0.05, 1.04)	0.13 (0.01, 0.53)	0.68 (0.22, 1.45)	<0.001
2‐h postprandial C‐peptide (M [P_25_, P_75_], ng/mL)		0.92 (0.07, 2.63)	0.37 (0.01, 1.40)	1.68 (0.47, 3.48)	< 0.001

For demographic indicators, age, sex, and marital status were comparable between the two groups (all *p* ≥ 0.05), whereas statistically significant intergroup differences were detected in ethnicity, educational level, and geographic origin (all *p* < 0.05).

With regard to clinical parameters, body mass index (BMI), length of hospital stay, diabetes duration, prior hypoglycemia history, and insulin pump therapy showed no statistically significant between‐group differences (all *p* ≥ 0.05). In contrast, significant disparities existed in smoking and alcohol drinking histories (all *p* < 0.05).

In laboratory examinations, the largest amplitude of glycemic excursions (LAGE), fasting C‐peptide, and 2‐h postprandial C‐peptide (2 h‐CP) were comparable across cohorts (all *p* ≥ 0.05), whereas HbA1c differed significantly between groups (*p* < 0.05).

### 3.3. Univariate Analysis of Associated Factors for In‐Hospital Hypoglycemia in LADA Patients

Univariate logistic regression was performed based on predefined analytical protocols. Pooled effect estimates for variables significantly associated with in‐hospital hypoglycemia (*p* < 0.05) are summarized in Table 2. All univariate results are presented in Table S3. Variables with *p* < 0.1 in univariate analysis underwent multicollinearity assessment prior to multivariate regression.

**Table 2:** Univariate analysis of factors associated with in‐hospital hypoglycemia in patients with LADA (*p* < 0.05).

Variables	OR (95% CI)	*p* value
Geographic origin	0.357 (0.259–0.493)	< 0.001
Sex	0.637 (0.490–0.898)	0.008
Ethnicity	1.694 (1.216–2.360)	0.006
Educational level	0.652 (0.479–0.886)	0.013
Length of hospital stay	1.140 (1.091–1.190)	< 0.001
BMI	0.916 (0.871–0.964)	< 0.001
Diabetes duration	1.050 (1.024–1.076)	0.001
Prior hypoglycemia history	3.061 (2.052–4.566)	< 0.001
Diabetic peripheral neuropathy	1.537 (1.111–2.127)	0.010
Postprandial glucose excursion	0.946 (0.896–0.998)	0.044
LAGE	1.156 (1.111–1.204)	< 0.001
Insulin pump therapy	6.143 (3.308–11.407)	< 0.001
Fasting C‐peptide	0.511 (0.398–0.655)	< 0.001
2 h‐CP	0.785 (0.717–0.860)	< 0.001
Estimated glomerular filtration rate (eGFR)	0.991 (0.985–0.998)	0.008
Total cholesterol (TC)	0.845 (0.747–0.957)	0.008
Triglycerides (TG)	0.882 (0.778–0.999)	0.049
Low‐density lipoprotein cholesterol (LDL‐C)	0.706 (0.591–0.843)	< 0.001
Hemoglobin (Hb)	0.989 (0.981–0.996)	0.003

### 3.4. Multicollinearity Screening of Candidate Variables

All variables retained after univariate screening (*p* < 0.1) underwent multicollinearity evaluation. The average VIF of candidate variables ranged from 1.059 to 3.919 (Table 3), with no average VIF exceeding the predefined cutoff of 10, indicating the absence of severe multicollinearity. Consistent with the VIF results, the correlation heatmap (Figure S1), generated from average correlation coefficients across 20 imputed datasets, revealed weak pairwise associations among variables. No pairs of highly correlated variables required exclusion, and all screened variables were retained for subsequent multivariate logistic regression analysis.

**Table 3:** Average VIF of candidate variables after multiple imputation.

Variables	VIF
TC	3.919
LDL‐C	3.400
2 h‐CP	2.612
Fasting C‐peptide	2.586
Geographic origin	1.553
Hb	1.488
HbA1c	1.432
Triglycerides (TG)	1.404
Diabetes duration	1.323
Sex	1.312
BMI	1.270
Ethnicity	1.269
eGFR	1.258
Diabetic peripheral neuropathy	1.201
LAGE	1.165
Prior hypoglycemia history	1.163
Length of hospital stay	1.153
Insulin pump therapy	1.111
Educational level	1.104
White blood cell count	1.097
Postprandial glucose excursion	1.088
Serum potassium	1.077
Use of oral antidiabetic agents	1.059

### 3.5. Binary Logistic Regression Analysis of Factors Associated With In‐Hospital Hypoglycemia in Patients With LADA

In‐hospital hypoglycemia was defined as the binary dependent variable (0 = absent, 1 = present). All variables screened via univariate analysis (*p* < 0.1) and free of severe multicollinearity were included in the multivariate model. Continuous variables were retained in original forms, and categorical variables were converted into dummy variables with predefined reference categories. Detailed variable coding is presented in Table 4.

**Table 4:** Coding assignment of independent variables.

Independent variables	Coding assignment
Geographic origin	0 = Yunnan Province, 1 = Chongqing Municipality
Sex	0 = male, 1 = female
Prior hypoglycemia history	0 = no, 1 = yes
Diabetic peripheral neuropathy	0 = no, 1 = yes
Use of oral antidiabetic agents	0 = no, 1 = yes
Insulin pump therapy	0 = no, 1 = yes
Educational level	0 = below senior high school, 1 = senior high school and above
Ethnicity	0 = Han ethnicity, 1 = ethnic minorities

Regression analyses were performed on each of the 20 multiply imputed datasets, and final effect estimates were pooled in accordance with Rubin′s rules. Multiple independently associated factors for in‐hospital hypoglycemia were identified (*p* < 0.05), including insulin pump therapy (OR = 8.233, 95% CI: 3.914–17.274), the LAGE (OR = 1.136, 95% CI: 1.081–1.193), length of hospital stay (OR = 1.134, 95% CI: 1.074–1.197), geographic origin (OR = 0.369, 95% CI: 0.225–0.605), prior hypoglycemia history (OR = 2.447, 95% CI: 1.448–4.024), HbA1c (OR = 0.902, 95% CI: 0.837–0.973), and sex (OR = 1.655, 95% CI: 1.065–2.574). Relevant OR values, 95% CIs and *p* values are summarized in Table 5.

**Table 5:** Binary logistic regression analysis of factors associated with in‐hospital hypoglycemia in patients with LADA (*n* = 709).

Variables	B	SE	*p* value	OR (95% CI)
Length of hospital stay	0.126	0.028	< 0.001	1.134 (1.074–1.197)
Largest amplitude of glycemic excursions	0.127	0.025	< 0.001	1.136 (1.081–1.193)
Glycated hemoglobin	−0.103	0.038	0.007	0.902 (0.837–0.973)
Geographic origin	−0.997	0.253	< 0.001	0.369 (0.225–0.605)
Sex	0.504	0.225	0.025	1.655 (1.065–2.574)
Insulin pump therapy	2.107	0.379	< 0.001	8.233 (3.914–17.274)
Prior hypoglycemia history	0.895	0.254	< 0.001	2.447 (1.448–4.024)

### 3.6. Model Performance and Sensitivity Analysis

Discriminative efficiency and goodness of fit indicators were calculated for each imputed dataset. The median AUC reached 0.836 (range: 0.831–0.845), demonstrating favorable discriminative capacity to differentiate patients with or without in‐hospital hypoglycemia. At the median optimal cut‐off value of 0.466 (range: 0.374–0.519), the median sensitivity and specificity were 0.780 (range: 0.723–0.857) and 0.757 (range: 0.673–0.810), reflecting balanced diagnostic performance of the established model.

Regarding explanatory power, the median McFadden *R* ^2^, Cox–Snell *R* ^2^, and Nagelkerke *R* ^2^ were 0.275 (0.266–0.289), 0.316 (0.308–0.330), and 0.422 (0.412–0.440). These *R* ^2^ values indicated that the screened associated factors exhibited moderate explanatory capacity for in‐hospital hypoglycemia onset among LADA patients.

Sensitivity analysis was conducted to examine the robustness regarding the number of imputed datasets. Ten datasets were randomly sampled from the full imputed pool to refit the multivariate logistic regression containing all 23 candidate variables, and the derived outcomes were compared with estimates based on all 20 datasets. Absolute differences in OR values between the two analytic schemes were less than 3%, with a median difference of approximately 0.1%, and discrepancies in corresponding *p* values were negligible. ORs obtained from the two modeling strategies were nearly perfectly correlated (Pearson *r* = 0.99999, *p* < 0.001). Detailed comparison data are listed in Table S4. Taken together, these findings indicated that 20 imputations were sufficient to produce stable parameter estimates, and the core conclusions were insensitive to variations in imputation quantity.

## 4. Discussion

LADA is characterized by insidious disease onset and adult‐onset pathogenic features, which complicate individualized glycemic management. Hypoglycemia represents a notable adverse effect of insulin‐based regimens and frequently complicates inpatient management of LADA. It triggers repeated therapeutic revisions and worsens multimorbid conditions, making hypoglycemia risk the core outcome assessed in the present study [26]. Globally, real‐world data on in‐hospital hypoglycemia among LADA inpatients remain scarce, especially from multiethnic regions of Southwest China, highlighting the clinical value of this multicenter investigation.

### 4.1. High Prevalence of In‐Hospital Hypoglycemic Events Among Hospitalized LADA Patients in Southwest China

Based on retrospective multicenter data from five tertiary hospitals across Southwest China, this study demonstrated a high pooled hypoglycemia prevalence of 47.39% and identified seven independently associated factors for in‐hospital hypoglycemia. These region‐specific findings support the development of localized hypoglycemia prevention strategies for LADA patients in the region.

As a hybrid diabetic phenotype with pathological characteristics of Type 1 and Type 2 diabetes, LADA creates inherent challenges for glycemic control. Uneven distribution of medical resources and diverse ethnic dietary habits across Southwest China further impede standardized hypoglycemia management. Collectively, the results fill regional epidemiological gaps and provide practical insights to alleviate the burden of hypoglycemia in this understudied population.

### 4.2. Independent Associated Factors for In‐Hospital Hypoglycemia in Patients With LADA

#### 4.2.1. Geographic Disparity as an Independent Associated Factor

Geographic origin emerged as an independently associated factor for hypoglycemia risk. The hypoglycemia incidence reached 56.44% in patients from Yunnan, nearly twice the rate of 31.66% observed in Chongqing, implying that inter‐regional environmental and healthcare disparities modify hypoglycemia susceptibility.

Yunnan′s elevated hypoglycemia risk is partly attributable to distinct regional characteristics documented in previous studies. Previous studies associated local ethnic dietary preferences for high‐glycemic‐index carbohydrates, irregular meal patterns, and insufficient standardized LADA management in primary care settings with inappropriate treatment regimens [27, 28]. In contrast, the mature healthcare system in Chongqing facilitates standardized diagnosis and management of LADA, which correlates with better adherence to medications and dietary guidance and a lower risk of hypoglycemia [29].

Such interregional differences contribute to heterogeneous hypoglycemia risk among enrolled inpatients. Clinically, stratified regional prevention strategies are warranted. Yunnan may benefit from prioritizing primary‐care clinician training and population‐level dietary interventions. Future prospective studies may incorporate regional dietary and healthcare indicators to explore modifiable factors associated with hypoglycemia.

#### 4.2.2. Baseline HbA1c and Its Inverse Association with In‐hospital Hypoglycemia Risk

HbA1c is inversely associated with in‐hospital hypoglycemia risk, which seemingly contradicts the conventional consensus that intensive glycemic control increases hypoglycemia risk [30]. The median baseline HbA1c of the cohort was 10.5%, indicating a high prevalence of chronic hyperglycemia. In the context of markedly elevated baseline glycemia, clinicians tend to adopt conservative insulin titration strategies to avoid rapid glycemic decline, thereby reducing hypoglycemia occurrence in patients with high HbA1c [26].

This inverse association should not be interpreted as a protective effect of elevated HbA1c against hypoglycemia, owing to reverse causality and inherent limitations of HbA1c testing. Persistent severe hyperglycemia may mask asymptomatic hypoglycemia and lead to underreporting of hypoglycemic events [31]. Furthermore, HbA1c only reflects months‐long average glycemia instead of acute glucose fluctuations, failing to capture transient asymptomatic hypoglycemia and consequently exaggerating the inverse association [32]. Therefore, this association arises from sustained hyperglycemia and conservative insulin administration, rather than a biological protective effect of elevated HbA1c.

This finding highlights the necessity of individualized glycemic targets for LADA patients. Uniform glycemic goals are inappropriate for this heterogeneous population, and dynamic adjustment of therapeutic thresholds should be guided by serial C‐peptide monitoring [33, 34].

#### 4.2.3. Length of Hospital Stay, Diabetes Duration, and Glycemic Fluctuation

Longer length of hospital stay synergistically increases hypoglycemia risk in patients with prolonged diabetes duration. Previous studies have confirmed that progressive *β*‐cell loss in long‐standing diabetes impairs physiological glucose counterregulation [35]. The present data further substantiate the synergistic adverse effect of disease chronicity and prolonged inpatient stay on hypoglycemia occurrence.

Long‐standing LADA is characterized by impaired residual islet function and diminished glycemic counterregulation [36]. Frequent adjustments to inpatient treatment regimens, combined with reduced physical activity and irregular dietary patterns during hospitalization, collectively elevate cumulative hypoglycemia risk. These factors compound baseline islet dysfunction and hospitalization‐related metabolic stress [37–39]. Closely associated with hospital stay, the LAGE reflects this cumulative glycemic risk. Clinicians are advised to comprehensively assess hypoglycemia risk according to both disease duration and length of hospitalization, and initiate early continuous glucose monitoring (CGM) to establish personalized glycemic profiles and optimize treatment strategies [40, 41].

#### 4.2.4. Insulin Pump Therapy and Interpretation of Indication Bias

Insulin pump therapy was associated with a substantially higher risk of hypoglycemia. This finding is primarily attributed to indication bias rather than inherent safety concerns regarding pump therapy. LADA presents heterogeneous *β*‐cell exhaustion and variable insulin sensitivity, which necessitates individualized basal‐bolus pump settings to achieve safe and stable glycemic control [42].

In clinical practice, insufficient assessment of residual endogenous islet function and infrequent adjustment of pump parameters disrupt insulin–glucose homeostasis. This problem is exacerbated by multiple physiological stressors during acute hospitalization [43]. Patients treated with insulin pumps in this cohort were high‐risk individuals with advanced islet impairment and unstable glycemia at baseline, which accounted for their greater susceptibility to hypoglycemia.

#### 4.2.5. Gender and Prior Hypoglycemia History

Female sex and prior hypoglycemia history are independently associated with in‐hospital hypoglycemia. Perimenopausal estrogen fluctuations alter insulin sensitivity and increase vulnerability to hypoglycemia in female patients [44], which is consistent with the mean age of 50.93 years among female participants in this cohort. Females also have higher symptom‐perception thresholds for hypoglycemia, leading to delayed rescue intervention [45]. A history of hypoglycemia indicates impaired pancreatic reserve, a key progressive pathological characteristic of LADA [46]. Hospitalization‐related stress, including infection, surgery, and medication adjustment, further disrupts glucose homeostasis and triggers recurrent hypoglycemia [47, 48]. Clinically, patients with a prior hypoglycemia history require intensified glucose monitoring and individualized therapeutic regimens [43, 47].

#### 4.2.6. Emerging Incretin‐Based Therapies for Hypoglycemia Risk Mitigation

Beyond conventional insulin‐centered regimens, GLP‐1RAs and dual GIP/GLP‐1 agonists offer insulin‐sparing therapeutic options to stabilize glycemic fluctuations and lower hypoglycemia risk. Post hoc analyses from the AWARD trial confirmed that dulaglutide achieves favorable glycemic control without increasing hypoglycemia incidence across varying GADA levels [18]. In patients with GADA‐positive LADA, tirzepatide produces significant reductions in HbA1c and body weight. Mild hypoglycemia may occur when tirzepatide is combined with insulin, whereas rational dose titration effectively prevents severe hypoglycemic episodes [17]. Although stratified medication guided by residual *β*‐cell function and autoantibody status is theoretically promising for individualized inpatient care to reduce insulin‐related hypoglycemia, relevant in‐hospital application criteria cannot be established due to limited real‐world evidence. In this context, the hypoglycemia associated factors identified in the present study can serve as core reference indicators to support the future standardized use of these incretin‐based agents among inpatients.

### 4.3. Limitations and Future Perspectives

This study has several limitations. First, data on potential confounders, including comorbidity grading and nutritional status, were not fully collected, which may compromise the accuracy of regression analyses. Second, all participants were recruited from tertiary hospitals across Southwest China. The distinct ethnic and healthcare characteristics of this region limit the generalizability of the findings to primary care populations and other geographic regions. Third, participants were not stratified by autoantibody profiles, and thus subtype‐specific hypoglycemia risk patterns were not explored. Fourth, the first in‐hospital hypoglycemic episode was defined as a binary endpoint. The database lacked detailed classification and characterization of hypoglycemia, which prevented the calculation of patient‐day hypoglycemia rates, time‐to‐first‐event analyses, severity assessments, and stratified analyses. Fifth, CGM was used infrequently, and glucose monitoring mainly relied on intermittent bedside capillary glucose testing. This approach may fail to capture transient, asymptomatic, and nocturnal hypoglycemia, thereby underestimating the overall hypoglycemia burden.

Future prospective multicenter studies covering different tiers of medical institutions are warranted to validate the present findings. Autoantibody stratification, expanded inpatient CGM application, and complete documentation of hypoglycemia classification and onset time would improve the accuracy of hypoglycemia prevalence estimates and enable diversified statistical analyses. Meanwhile, multiomics analyses can further clarify the molecular mechanisms underlying hypoglycemia in patients with LADA.

## 5. Conclusions

Based on multicenter retrospective data from five tertiary hospitals in Southwest China, this study systematically investigated the prevalence of in‐hospital hypoglycemia and its associated factors among hospitalized patients with LADA. The results revealed two key findings. First, the incidence of hypoglycemia in hospitalized LADA patients in Southwest China was notably high and exhibited distinct regional heterogeneity. Second, seven independently associated factors were identified, encompassing length of hospital stay, the LAGE, HbA1c, geographic origin, sex, insulin pump therapy, and prior hypoglycemia history.

These findings confer substantial academic and clinical value. Epidemiologically, they enrich the real‐world evidence regarding hypoglycemia among LADA patients residing in multiethnic regions of Southwest China. Theoretically, they expand the research framework of complication‐related studies focusing on this unique diabetes subtype. Clinically, they provide credible evidence for precise hypoglycemia risk stratification and the formulation of regionally tailored hypoglycemia prevention and management strategies.

Practically, these findings help improve inpatient safety, optimize long‐term prognosis, and promote the development of precision diabetes management in Southwest China.

Future research may adopt prospective multicenter designs combined with wearable glucose monitoring and multiomics techniques to further elucidate the molecular mechanisms underlying LADA‐related hypoglycemia and its interactions with regional environmental and dietary characteristics. Such efforts will facilitate the optimization of hypoglycemia risk prediction models and support the development of individualized prevention strategies for LADA patients.

## Author Contributions

**Haojie Zhou**: conceptualization, methodology, software, validation, formal analysis, investigation, resources, data curation, writing—original draft preparation, writing—review and editing, visualization, project administration, and funding acquisition; **Qiang Zhang**: methodology, software, validation, formal analysis, data curation, and writing—review and editing; **Guniqing Zhu**: methodology, validation, investigation, data curation, and writing—original draft preparation; **Weiwei Xu**: investigation and resources; **Xueyan Zhao**: resources; **Xiangfu Gu**: investigation; **Xiaoyu Cai**: investigation; **Xiaoli Zhu**: resources, supervision, and funding acquisition; **Ran Li**: investigation, resources, supervision, and funding acquisition. Haojie Zhou, Qiang Zhang, and Guniqing Zhu contributed equally to this article (co‐first authors).

## Funding

This study was supported by the Yunnan Provincial Department of Education Scientific Research Fund Project (2026Y1327), Science Popularization Special Project of the Innovation Guidance and Sci‐Tech Enterprise Cultivation Program, Bureau of Science and Technology of Dali Bai Autonomous Prefecture, Yunnan Province (20262904D040003), Kuanren Talents Program of the second affiliated hospital of Chongqing Medical University, and Chronic Disease Management Research Project of National Health Commission Capacity Building and Continuing Education Center (GWJJMB202510021079).

## Disclosure

All authors have read and approved the final version of the manuscript. Corresponding authors have full access to all of the data in this study and takes complete responsibility for the integrity of the data and the accuracy of the data analysis.

## Ethics Statement

The study was ethically approved by the Ethics Committee of the First Affiliated Hospital of Dali University (DFY20250520002) and the Ethics Committee of the Second Affiliated Hospital of Chongqing Medical University (275[2025]). Written informed consent was waived owing to the retrospective nature of this analysis, and all study procedures were performed in compliance with relevant national legal provisions and institutional regulatory requirements.

## Conflicts of Interest

The authors declare no conflicts of interest.

## Supporting Information

Additional supporting information can be found online in the Supporting Information section.

## Supporting information


**Supporting Information 1** Table S1: Distribution of missing values for all study variables. Table S2: Intergroup comparisons of all measured variables. Table S3: Full results of univariate logistic regression for hypoglycemia in hospitalized patients with LADA. Table S4: Sensitivity analysis of multivariate logistic regression across imputed datasets.

**Supporting Information 2** Figure S1: Heatmap of averaged correlation coefficients among screened variables derived from 20 multiply imputed datasets.

## Data Availability

The data that support the findings of this study are available on request from the corresponding authors. The data are not publicly available due to privacy or ethical restrictions.

## References

[1] Santoso C. , Wei Y. , Ahlqvist E. , Tuomi T. , and Carlsson S. , Autoimmune Diseases and the Risk and Prognosis of Latent Autoimmune Diabetes in Adults, Diabetologia. (2025) 68, no. 2, 331–341, 10.1007/s00125-024-06303-4. 39467873 PMC11732938

[2] Pozzilli P. and Pieralice S. , Latent Autoimmune Diabetes in Adults: Current Status and New Horizons, Endocrinology and Metabolism. (2018) 33, no. 2, 10.3803/EnM.2018.33.2.147. PMC6021307 29947172

[3] Manisha A. M. , Shangali A. R. , Mfinanga S. G. , and Mbugi E. V. , Prevalence and Factors Associated With Latent Autoimmune Diabetes in Adults (LADA): A Cross-Sectional Study, BMC Endocrine Disorders. (2022) 22, no. 1, 175–184, 10.1186/s12902-022-01089-1. 35804315 PMC9270809

[4] Lundholm M. D. and Zhou K. , Latent Autoimmune Diabetes in Adults: Not Type 1, Not Type 2, a Little of Both, Cleveland Clinic Journal of Medicine. (2025) 92, no. 12, 757–763, 10.3949/ccjm.92a.25069. 41326180

[5] Mo Y. , Zhao F. , Yuan L. , Xing Q. , Zhang M. , and Zhao C. , Consensus on Medical Protocol Prescriptions for Blood Glucose Monitoring in Adult In-Patients With Hyperglycemia, Chinese Journal of Nursing. (2019) 54, no. 8, 1142–1147, 10.3761/j.issn.0254-1769.2019.08.004.

[6] Eshetu K. , Regassa L. D. , Dehresa M. , and Genete D. , Chronic Microvascular Complication of Type 1 Diabetes Mellitus and Its Predictors Among Children With Type 1 Diabetes Mellitus in Ethiopia; a Single Center Experience: Ambi Directional Cohort Study, Pediatric Health, Medicine and Therapeutics. (2024) 15, 201–212, 10.2147/PHMT.S456541. 38808177 PMC11130991

[7] Sudhanshu S. , Nair V. V. , Godbole T. , Reddy S. V. B. , Bhatia E. , Dabadghao P. , Sharma K. , Arora P. , Bano S. , Singh A. , and Bhatia V. , Glycemic Control and Long-Term Complications in Pediatric Onset Type 1 Diabetes Mellitus: A Single-Center Experience From Northern India, Indian Pediatrics. (2019) 56, no. 3, 191–195, 10.1007/s13312-019-1497-3, 30954988. 30954988

[8] Amiel S. A. , The Consequences of Hypoglycaemia, Diabetologia. (2021) 64, no. 5, 963–970, 10.1007/s00125-020-05366-3. 33550443 PMC8012317

[9] Chen S. , Lu J. , Peng D. , Liu F. , Lu W. , Zhu W. , Bao Y. , Zhou J. , and Jia W. , Incidence Rate and Risk Factors for Hypoglycemia Among Individuals With Type 1 Diabetes or Type 2 Diabetes in China Receiving Insulin Treatment, Diabetes Research and Clinical Practice. (2023) 206, 110987, 10.1016/j.diabres.2023.110987, 37925076. 37925076

[10] Pisal T. , Gupta P. R. , Mestry D. B. , and Arke A. D. , Latent Autoimmune Diabetes in Adults: A Case Series and Clinical Insights, Cureus. (2025) 8, no. 17, e90879, 10.7759/cureus.90879. PMC12456252 40995267

[11] Pieralice S. and Pozzilli P. , Latent Autoimmune Diabetes in Adults: A Review on Clinical Implications and Management, Diabetes and Metabolism Journal. (2018) 42, no. 6, 451–464, 10.4093/dmj.2018.0190. 30565440 PMC6300440

[12] Chan J. C. , Elaine C. , Kong A. , Cheung E. , Lim C. , Fan B. , Tsoi S. , Fan Y. , Shi M. , Ozaki R. , Ma R. , and Luk A. , Multifaceted Nature of Young-Onset Diabetes - Can Genomic Medicine Improve the Precision of Diagnosis and Management?, Journal of Translational Genetics and Genomics. (2024) 8, no. 1, 13–34, 10.20517/jtgg.2023.36.

[13] Tomás G. V. , Diego R. O. , Alba G. H. , Alonso Felgueroso C. , Martínez Tamés G. , Lambert C. , Delgado-Álvarez E. , and Menéndez Torre E. , Hypoglycemia in Patients With Type 2 Diabetes Mellitus During Hospitalization: Associated Factors and Prognostic Value, Diabetology & Metabolic Syndrome. (2023) 15, no. 1, 249–249, 10.1186/s13098-023-01212-9, 38044455. 38044455 PMC10694969

[14] Tang L. , Chang S. J. , Chen C. J. , and Liu J. T. , Non-Invasive Blood Glucose Monitoring Technology: A Review, Sensors. (2020) 20, no. 23, 10.3390/s20236925. PMC7731259 33291519

[15] Amiel S. A. , Aschner P. , Childs B. , Cryer P. E. , de Galan B. E. , Frier B. M. , Gonder-Frederick L. , Heller S. R. , Jones T. , Khunti K. , Leiter L. A. , Luo Y. , McCrimmon R. J. , Pedersen-Bjergaard U. , Seaquist E. R. , and Zoungas S. , Hypoglycaemia, Cardiovascular Disease, and Mortality in Diabetes: Epidemiology, Pathogenesis, and Management, Lancet Diabetes and Endocrinology. (2019) 7, no. 5, 385–396, 10.1016/S2213-8587(18)30315-2. 30926258

[16] Buzzetti R. , Tuomi T. , Mauricio D. , Pietropaolo M. , Zhou Z. , Pozzilli P. , and Leslie R. D. , Management of Latent Autoimmune Diabetes in Adults: A Consensus Statement From an International Expert Panel, Diabetes. (2020) 69, no. 10, 2037–2047, 10.2337/dbi20-0017, 32847960. 32847960 PMC7809717

[17] Peters A. L. , Buzzetti R. , Lee C. J. , Pavo I. , Liu M. , Karanikas C. A. , and Paik J. S. , Improved HbA1c and Body Weight in GADA-Positive Individuals Treated With Tirzepatide: A Post Hoc Analysis of SURPASS, Journal of Clinical Endocrinology and Metabolism. (2025) 110, no. 4, e962–e969, 10.1210/clinem/dgae372, 38824910. 38824910 PMC11913103

[18] Pozzilli P. , Leslie R. D. , Peters A. L. , Buzzetti R. , Shankar S. S. , Milicevic Z. , Pavo I. , Lebrec J. , Martin S. , and Schloot N. C. , Dulaglutide Treatment Results in Effective Glycaemic Control in Latent Autoimmune Diabetes in Adults (LADA): Apost‐hocanalysis of the AWARD-2, -4 and -5 Trials, Diabetes, Obesity and Metabolism. (2018) 20, no. 6, 1490–1498, 10.1111/dom.13237. 29377522

[19] Von Elm E. , Altman D. G. , Egger M. , Pocock S. J. , Gøtzsche P. C. , and Vandenbroucke J. P. , The Strengthening the Reporting of Observational Studies in Epidemiology (STROBE) Statement: Guidelines for Reporting Observational Studies, Journal of Clinical Epidemiology. (2008) 61, no. 4, 344–349, 10.1016/j.jclinepi.2007.11.008. 18313558

[20] Chinese Endocrinologist Association and National Clinical Research Center For Metabolic Diseases , China Experts′ Consensus on the Diagnosis and Treatment of Latent Autoimmune Diabetes in Adults (2021 Edition), National Medical Journal of China. (2021) 101, no. 38, 3077–3091, 10.3760/cma.j.cn112137-20210629-01463.

[21] American Diabetes Association , 2. Classification and Diagnosis of Diabetes: Standards of Medical Care in Diabetes—2018, Diabetes Care. (2018) 41, no. supplement 1, S13–S27, 10.2337/dc18-S002, 29222373. 29222373

[22] Sterne J. A. C. , White I. R. , Carlin J. B. , Spratt M. , Royston P. , Kenward M. G. , Wood A. M. , and Carpenter J. R. , Multiple Imputation for Missing Data in Epidemiological and Clinical Research: Potential and Pitfalls, BMJ. (2009) 338, b2393, 10.1136/bmj.b2393, 19564179. 19564179 PMC2714692

[23] Groenwold R. H. H. , Donders A. R. T. , Roes K. C. B. , Harrell F. E. , and Moons K. G. M. , Dealing With Missing Outcome Data in Randomized Trials and Observational Studies, American Journal of Epidemiology. (2012) 175, no. 3, 211–217, 10.1093/aje/kwr302. 22262640

[24] Stekhoven D. J. and Bühlmann P. , Miss Forest—Non-Parametric Missing Value Imputation for Mixed-Type Data, Bioinformatics. (2012) 28, no. 1, 112–118, 10.1093/bioinformatics/btr597. 22039212

[25] Buuren S. V. and Groothuis-Oudshoorn K. , mice: Multivariate Imputation by Chained Equations in R, Journal of Statistical Software. (2011) 45, no. 3, 1–67, 10.18637/jss.v045.i03.

[26] Zhou Z. , Xu M. , Xiong P. , Yuan J. , Zheng D. , and Piao S. , Prognosis and Outcome of Latent Autoimmune Diabetes in Adults: T1DM or T2DM?, Diabetology & Metabolic Syndrome. (2024) 16, no. 1, 242–250, 10.1186/s13098-024-01479-6. 39375804 PMC11457386

[27] Zhang Q. , Liu Z. , Hu W. , Chen X. , Li J. , Wan Q. , Zhao J. , Ruan Y. , Dao B. , Li Y. , and Min X. , Social Capital and Dietary Patterns in Three Ethnic Minority Groups Native to Yunnan Province, Southwest China, PLOS One. (2021) 16, no. 8, e0256078, 10.1371/journal.pone.0256078, 34383859. 34383859 PMC8360576

[28] Chen R. , Zhu W. , Wu Y. , Zhang H. W. , Yang M. R. , Liu Z. H. , Li Y. J. , Li Y. , Liu J. W. , and Han R. , Prevalence Rate of Type 2 Diabetes Mellitus and Related Influencing Factors in the Yi Population in Jinning District, Yunnan Province, China, Journal of Chongqing Medical University. (2025) 50, no. 1, 72–79, 10.13406/j.cnki.cyxb.003697.

[29] Quiroz-Aldave J. E. , Durand-Vásquez M. D. C. , Ildefonso-Najarro S. P. , Gamarra-Osorio E. R. , Puelles-León S. L. , Quesquén-García C. M. , Concepción-Urteaga L. A. , Paz-Ibarra J. , Alvarado-León E. , Urbina E. W. , and Herrera A. A. , The diagnosis of LADA diabetes in a low-resource country: a peruvian case series, Electronic Journal of General Medicine. (2025) 22, no. 4, 10.29333/ejgm/16301.

[30] Yamada T. , Shojima N. , Noma H. , Yamauchi T. , and Kadowaki T. , Glycemic Control, Mortality, and Hypoglycemia in Critically Ill Patients: A Systematic Review and Network Meta-Analysis of Randomized Controlled Trials, Intensive Care Medicine. (2017) 43, no. 1, 1–15, 10.1007/s00134-016-4523-0. 27637719

[31] Rickels M. R. , Hypoglycemia-Associated Autonomic Failure, Counterregulatory Responses, and Therapeutic Options in Type 1 Diabetes, Annals of the New York Academy of Sciences. (2019) 1454, no. 1, 68–79, 10.1111/nyas.14214. 31389033 PMC6945804

[32] Perlman J. E. , Gooley T. A. , McNulty B. , Meyers J. , and Hirsch I. B. , HbA1c and Glucose Management Indicator Discordance: A Real-World Analysis, Diabetes Technology & Therapeutics. (2021) 23, no. 4, 253–258, 10.1089/dia.2020.0501. 33253015 PMC8255314

[33] Stimson R. H. , Dover A. R. , Clarke C. , Conceicao C. , McDonald L. , Miller L. , Wise H. , Forbes S. , Wright R. J. , Lyall M. J. , Strachan M. W. J. , and Gibb F. W. , Persistent C-Peptide Secretion Is Associated With Favourable CGM Metrics in Adults With Type 1 Diabetes, Diabetologia. (2026) 69, no. 1, 59–68, 10.1007/s00125-025-06578-1, 41165798. 41165798 PMC12686044

[34] Gibb F. W. , McKnight J. A. , Clarke C. , and Strachan M. W. J. , Preserved C-Peptide Secretion Is Associated With Fewer Low-Glucose Events and Lower Glucose Variability on Flash Glucose Monitoring in Adults With Type 1 Diabetes, Diabetologia. (2020) 63, no. 5, 906–914, 10.1007/s00125-020-05099-3. 32034440 PMC7145780

[35] Gubitosi-Klug R. A. , Braffett B. H. , Hitt S. , Arends V. , Uschner D. , Jones K. , Diminick L. , Karger A. B. , Paterson A. D. , Roshandel D. , Marcovina S. , Lachin J. M. , Steffes M. , Palmer J. P. , and DCCT/EDIC Research Group , Residual *β* Cell Function in Long-Term Type 1 Diabetes Associates With Reduced Incidence of Hypoglycemia, Journal of Clinical Investigation. (2021) 131, no. 3, e143011, 10.1172/JCI143011, 33529168. 33529168 PMC7843223

[36] Joseph J. , Latent Autoimmune Diabetes in Adults: Diagnostic and Therapeutic Challenges, Cureus. (2025) 17, no. 9, e92356, 10.7759/cureus.92356. 41103892 PMC12522475

[37] Tsur A. , Cahn A. , Israel M. , Feldhamer I. , Hammerman A. , and Pollack R. , Impact of Flash Glucose Monitoring on Glucose Control and Hospitalization in Type 1 Diabetes: A Nationwide Cohort Study, Diabetes/Metabolism Research and Reviews. (2021) 37, no. 1, e3355, 10.1002/dmrr.3355. 32469094

[38] Flanagan D. , Avari P. , Choudhary P. , Lumb A. , Misra S. , Rayman G. , and Dhatariya K. , Using Technology to Improve Diabetes Care in Hospital: The Challenge and the Opportunity, Journal of Diabetes Science and Technology. (2023) 17, no. 2, 503–508, 10.1177/19322968221138299, 36433805. 36433805 PMC10012371

[39] Kashiwagi K. , Inaishi J. , Kinoshita S. , Wada Y. , Hanashiro S. , Shiga K. , Kitazawa M. , Tsutsumi S. , Yamakawa H. , Irie J. , and Kishimoto T. , Assessment of Glycemic Variability and Lifestyle Behaviors in Healthy Nondiabetic Individuals According to the Categories of Body Mass Index, PLOS One. (2023) 18, no. 10, e0291923, 10.1371/journal.pone.0291923, 37792730. 37792730 PMC10550127

[40] Parise M. , Di Molfetta S. , Graziano R. T. , Fiorentino R. , Cutruzzolà A. , Gnasso A. , and Irace C. , A Head-to-Head Comparison of Two Algorithms for Adjusting Mealtime Insulin Doses Based on CGM Trend Arrows in Adult Patients With Type 1 Diabetes: Results From an Exploratory Study, International Journal of Environmental Research and Public Health. (2023) 20, no. 5, 10.3390/ijerph20053945, 36900956. PMC10002216 36900956

[41] Aleppo G. , Laffel L. M. , Ahmann A. J. , Hirsch I. B. , Kruger D. F. , Peters A. , Weinstock R. S. , and Harris D. R. , A Practical Approach to Using Trend Arrows on the Dexcom G5 CGM System for the Management of Adults With Diabetes, Journal of the Endocrine Society. (2017) 1, no. 12, 1445–1460, 10.1210/js.2017-00388, 29344577. 29344577 PMC5760210

[42] Biester T. , Berget C. , Boughton C. , Cudizio L. , Ekhlaspour L. , Hilliard M. E. , Reddy L. , Sap Ngo Um S. , Schoelwer M. , Sherr J. L. , and Dovc K. , International Society for Pediatric and Adolescent Diabetes Clinical Practice Consensus Guidelines 2024: Diabetes Technologies - Insulin Delivery, Hormone Research in Paediatrics. (2025) 97, no. 6, 636–662, 10.1159/000543034, 39657603. PMC11854989 39657603

[43] Pasquel F. J. , Lansang M. C. , Dhatariya K. , and Umpierrez G. E. , Management of Diabetes and Hyperglycaemia in the Hospital, Lancet Diabetes & Endocrinology. (2021) 9, no. 3, 174–188, 10.1016/S2213-8587(20)30381-8, 33515493. 33515493 PMC10423081

[44] Bansal S. , Balagopalan J. P. , Dani S. , Zargar A. H. , Bhattacharyya A. , Deka N. , Taraphder A. , Almeida A. , Jain S. , and Swami O. C. , Diabetes and Cardiovascular Risk in Menopausal Women: An Emerging Concern, European Journal of Cardiovascular Medicine. (2025) 15, no. 7, 246–257, 10.5083/ejcm/25-07-46.

[45] Lin Y. K. , Agni A. , Chuisano S. , de Zoysa N. , Fetters M. , Amiel S. A. , Pop-Busui R. , and DeJonckheere M. , You Have to Use Everything and Come to Some Equilibrium: A Qualitative Study on Hypoglycemia Self-Management in Users of Continuous Glucose Monitor With Diverse Hypoglycemia Experiences, BMJ Open Diabetes Research & Care. (2023) 11, no. 3, e003415, 10.1136/bmjdrc-2023-003415, 37349107. PMC10314535 37349107

[46] Nishimura A. , Matsumura K. , Kikuno S. , Nagasawa K. , Okubo M. , Mori Y. , and Kobayashi T. , Slowly Progressive Type 1 Diabetes Mellitus: Current Knowledge and Future Perspectives, Diabetes, Metabolic Syndrome and Obesity: Targets and Therapy. (2019) 12, 2461–2477, 10.2147/DMSO.S191007, 31819572. 31819572 PMC6886592

[47] Herranz-Antolín S. , Cotón-Batres C. , López-Virgos M. C. , Esteban-Monge V. , Álvarez-De Frutos V. , and Torralba M. , Factors Associated With Glycemia Risk Index in a Cohort of Patients With Type 1 Diabetes Mellitus and Latent Autoimmune Diabetes in Adults (LADA), Endocrine. (2024) 86, no. 2, 574–583, 10.1007/s12020-024-03901-5. 38844609

[48] Mitsuyama Y. , Shimizu K. , Komukai S. , Hirayama A. , Takegawa R. , Ebihara T. , Kitamura T. , Ogura H. , and Shimazu T. , Sepsis-Associated Hypoglycemia on Admission Is Associated With Increased Mortality in Intensive Care Unit Patients, Acute Medicine & Surgery. (2022) 9, no. 1, e718, 10.1002/ams2.718, 35106180. 35106180 PMC8785236

