# Supplementary material for: Risk of Hypoglycemia and Associated Factors Among In‐Hospital Chinese Patients With Latent Autoimmune Diabetes in Adults (LADA): A Multicenter Retrospective Cohort Study
Source: J Diabetes Res. 2026 Jun 29;2026:2961800. doi: 10.1155/jdr/2961800 (PMC13312302; doi:10.1155/jdr/2961800)
Supplement: Supplementary file 1 — Supporting Information 1 Table S1: Distribution of missing values for all study variables. Table S2: Intergroup comparisons of all measured variables. Table S3: Full results of univariate logistic regression for hypoglycemia in hospitalized patients with LADA. Table S4: Sensitivity analysis of multivariate logistic regression across imputed datasets. [file JDR-2026-2961800-s001.docx]

Table S1 Distribution of missing values for all study variables

| **Variables** | **Valid cases**  **(*n*)** | **Missing count**  **(*n*)** | **Missing rate**  **(%)** | **Variables** | **Valid cases**  **(*n*)** | **Missing count**  **(*n*)** | **Missing rate**  **(%)** |
| --- | --- | --- | --- | --- | --- | --- | --- |
| Age (years) | 709 | 0 | 0.00 | Hypoglycemia occurrence | 709 | 0 | 0.00 |
| Body mass index (kg/m²) | 681 | 28 | 3.95 | Geographic origin | 709 | 0 | 0.00 |
| Length of hospital stay (days) | 709 | 0 | 0.00 | Sex | 709 | 0 | 0.00 |
| Smoking duration (years) | 700 | 9 | 1.27 | Marital status | 709 | 0 | 0.00 |
| Alcohol drinking duration (years) | 702 | 7 | 0.99 | Ethnicity | 709 | 0 | 0.00 |
| Age at diabetes onset (years) | 709 | 0 | 0.00 | Educational level | 709 | 0 | 0.00 |
| Diabetes duration (years) | 709 | 0 | 0.00 | Smoking history | 709 | 0 | 0.00 |
| Total daily insulin dosage (IU/d) | 679 | 30 | 4.23 | Alcohol drinking history | 709 | 0 | 0.00 |
| Preprandial insulin dosage (IU/d) | 678 | 31 | 4.37 | Prior hypoglycemia history | 709 | 0 | 0.00 |
| Long-acting insulin dosage (IU/d) | 678 | 31 | 4.37 | Family history of diabetes | 709 | 0 | 0.00 |
| Postprandial glucose excursion (mmol/L) | 503 | 206 | 29.06 | History of hypertension | 709 | 0 | 0.00 |
| Largest amplitude of glycemic excursions (mmol/L) | 701 | 8 | 1.13 | Pre-existing chronic heart disease | 709 | 0 | 0.00 |
| Mean in-hospital blood glucose (mmol/L) | 709 | 0 | 0.00 | Diabetic ketosis | 709 | 0 | 0.00 |
| Glycated hemoglobin (%) | 693 | 16 | 2.26 | Diabetic ketoacidosis | 709 | 0 | 0.00 |
| Fasting C-peptide (ng/mL) | 634 | 75 | 10.58 | Hyperosmolar hyperglycemic state | 709 | 0 | 0.00 |
| 0.5-hour postprandial C-peptide (ng/mL) | 418 | 291 | 41.04 | Diabetic retinopathy | 709 | 0 | 0.00 |
| 1-hour postprandial C-peptide (ng/mL) | 445 | 264 | 37.24 | Diabetic nephropathy | 709 | 0 | 0.00 |
| 2-hour postprandial C-peptide (ng/mL) | 512 | 197 | 27.79 | Diabetic peripheral neuropathy | 709 | 0 | 0.00 |
| Serum creatinine (μmol/L) | 702 | 7 | 0.99 | Diabetic macrovascular complications | 709 | 0 | 0.00 |
| Estimated glomerular filtration rate (mL/min/1.73m²) | 702 | 7 | 0.99 | Diabetic foot | 709 | 0 | 0.00 |
| Total serum protein (g/L) | 678 | 31 | 4.37 | Sleep disturbance | 709 | 0 | 0.00 |
| Total cholesterol (mmol/L) | 688 | 21 | 2.96 | Polypharmacy | 709 | 0 | 0.00 |
| Triglyceride (mmol/L) | 688 | 21 | 2.96 | Use of oral antidiabetic agents | 709 | 0 | 0.00 |
| Low-density lipoprotein cholesterol (mmol/L) | 687 | 22 | 3.10 | Continuous glucose monitoring application | 709 | 0 | 0.00 |
| High-density lipoprotein cholesterol (mmol/L) | 687 | 22 | 3.10 | Insulin pump therapy | 709 | 0 | 0.00 |
| Hemoglobin (g/L) | 703 | 6 | 0.85 | Multiple insulin formulations application | 709 | 0 | 0.00 |
| Red blood cell count (×10^12^/L) | 702 | 7 | 0.99 | Glutamic acid decarboxylase antibody | 437 | 272 | 38.36 |
| White blood cell count (×10^9^/L) | 702 | 7 | 0.99 | Zinc transporter 8 antibody | 205 | 504 | 71.09 |
| C-reactive protein (mg/L) | 435 | 274 | 38.65 | Insulin autoantibody | 439 | 270 | 38.08 |
| 25-hydroxyvitamin D (ng/mL) | 284 | 425 | 59.94 | Insulinoma-Associated Antigen 2 Antibody | 205 | 504 | 71.09 |
| Urinary microalbumin (mg/L) | 405 | 304 | 42.88 | Islet cell antibody | 192 | 517 | 72.92 |
| Serum potassium (mmol/L) | 641 | 68 | 9.59 | Serum calcium (mmol/L) | 641 | 68 | 9.59 |
| Serum sodium (mmol/L) | 641 | 68 | 9.59 |  |  |  |  |

Table S2 Intergroup comparisons of all measured variables

| **Variables** | | **Total**  **(*n* = 709)** | **Hypoglycemia group**  **(*n* = 336)** | **Non-hypoglycemia group**  **(*n* = 373)** | ***p* value** |
| --- | --- | --- | --- | --- | --- |
| Age [$\bar{x}\pm s$, year] | | 50.24 ± 14.28 | 50.92 ± 13.60 | 49.62 ± 14.87 | 0.241 |
| Smoking duration [$\bar{x}\pm s$, year] | | 11.44 ± 14.51 | 10.73 ± 13.90 | 12.08 ± 15.04 | 0.219 |
| Age at diabetes onset [$\bar{x}\pm s$, year] | | 44.51 ± 13.78 | 44.28 ± 13.53 | 44.72 ± 14.02 | 0.674 |
| Total daily insulin dosage [$\bar{x}\pm s$, IU/d] | | 32.23 ± 14.10 | 31.65 ± 12.72 | 32.75 ± 15.24 | 0.300 |
| Preprandial insulin dosage [$\bar{x}\pm s$, IU/d] | | 19.71 ± 10.96 | 19.05 ± 10.35 | 20.31 ± 11.45 | 0.135 |
| Long-acting insulin dosage [$\bar{x}\pm s$, IU/d] | | 12.52 ± 7.49 | 12.51 ± 6.58 | 12.53 ± 8.23 | 0.974 |
| Postprandial glucose excursion [$\bar{x}\pm s$, mmol/L] | | 0.49 ± 3.10 | 0.21 ± 3.39 | 0.74 ± 2.80 | 0.046 |
| Largest amplitude of glycemic excursions [$\bar{x}\pm s$, mmol/L] | | 7.96 ± 4.38 | 9.29 ± 4.81 | 6.77 ± 3.56 | < 0.001 |
| Mean in-hospital blood glucose [$\bar{x}\pm s$, mmol/L] | | 9.75 ± 2.39 | 9.81 ± 2.30 | 9.70 ± 2.46 | 0.563 |
| Glycated hemoglobin [$\bar{x}\pm s$, %] | | 10.94 ± 3.11 | 10.73 ± 2.97 | 11.14 ± 3.22 | 0.090 |
| Estimated glomerular filtration rate [$\bar{x}\pm s$, mL/min/1.73m²] | | 104.07 ± 23.20 | 101.60 ± 22.35 | 106.30 ± 23.75 | 0.007 |
| Total serum protein [$\bar{x}\pm s$, g/L] | | 65.83 ± 8.95 | 65.49 ± 9.39 | 66.13 ± 8.54 | 0.368 |
| Low-density lipoprotein cholesterol [$\bar{x}\pm s$, mmol/L] | | 2.56 ± 0.95 | 2.40 ± 0.89 | 2.70 ± 0.98 | < 0.001 |
| Hemoglobin [$\bar{x}\pm s$, g/L] | | 139.08 ± 20.12 | 136.69 ± 20.03 | 141.24 ± 19.98 | 0.003 |
| Serum sodium [$\bar{x}\pm s$, mmol/L] | | 138.47 ± 4.08 | 138.63 ± 4.18 | 138.32 ± 3.99 | 0.333 |
| Body mass index [M (P_25_, P_75_), kg/m²] | | 21.38 (19.43, 23.48) | 20.82 (18.93, 22.89) | 21.72 (19.95, 23.91) | < 0.001 |
| Length of hospital stay [M (P_25_, P_75_), day] | | 9.00 (7.00, 12.00) | 10.00 (8.00, 13.00) | 8.00 (7.00, 10.00) | < 0.001 |
| Alcohol drinking duration [M (P_25_, P_75_), years] | | 0.00 (0.00, 3.60) | 0.00 (0.00, 0.00) | 0.00 (0.00, 8.00) | 0.380 |
| Diabetes duration [M (P_25_, P_75_), years] | | 4.00 (0.00, 9.00) | 6.00 (1.00, 10.00) | 2.00 (0.00, 7.00) | < 0.001 |
| Fasting C-peptide [M (P_25_, P_75_), ng/mL] | | 0.37 (0.05, 1.04) | 0.13 (0.01, 0.53) | 0.68 (0.22, 1.45) | < 0.001 |
| 2-hour postprandial C-peptide [M (P_25_, P_75_), ng/mL] | | 0.92 (0.07, 2.63) | 0.37 (0.01, 1.40) | 1.68 (0.47, 3.48) | < 0.001 |
| Serum creatinine [M (P_25_, P_75_), μmol/L] | | 61.73 (51.56, 74.79) | 62.05 (52.00, 74.42) | 61.15 (50.85, 74.88) | 0.546 |
| Total cholesterol [M (P_25_, P_75_), mmol/L] | | 4.43 (3.76, 5.30) | 4.32 (3.72, 5.01) | 4.57 (3.79, 5.54) | 0.005 |
| Triglyceride [M (P_25_, P_75_), mmol/L] | | 1.11 (0.79, 1.72) | 1.04 (0.76, 1.48) | 1.25 (0.83, 1.84) | < 0.001 |
| High-density lipoprotein cholesterol [M (P_25_, P_75_), mmol/L] | | 1.29 (1.04, 1.59) | 1.38 (1.10, 1.65) | 1.19 (1.00, 1.47) | < 0.001 |
| Red blood cell count [M (P_25_, P_75_), ×10^12^/L] | | 4.68 (4.26, 5.11) | 4.61 (4.20, 4.96) | 4.73 (4.33, 5.20) | 0.002 |
| White blood cell count [M (P_25_, P_75_), ×10^9^/L] | | 6.25 (5.03, 7.77) | 6.19 (4.93, 7.73) | 6.32 (5.20, 7.83) | 0.086 |
| Serum potassium [M (P_25_, P_75_), mmol/L] | | 4.05 (3.79, 4.30) | 4.08 (3.81, 4.33) | 4.02 (3.78, 4.26) | 0.108 |
| Serum calcium [M (P_25_, P_75_), mmol/L] | | 2.26 (2.16, 2.38) | 2.24 (2.14, 2.36) | 2.27 (2.17, 2.39) | 0.007 |
| Geographic origin | Yunnan Province | 450 (63.5%) | 254 (75.6%) | 196 (52.5%) | < 0.001 |
|  | Chongqing Municipality | 259 (36.5%) | 82 (24.4%) | 177 (47.5%) |  |
| Sex | Male | 429 (60.5%) | 186 (55.4%) | 243 (65.1%) | 0.010 |
|  | Female | 280 (39.5%) | 150 (44.6%) | 130 (34.9%) |  |
| Marital status | Unmarried | 59 (8.3%) | 23 (6.8%) | 36 (9.7%) | 0.335 |
|  | Married | 589 (83.1%) | 279 (83.0%) | 310 (83.1%) |  |
|  | Divorced | 47 (6.6%) | 26 (7.7%) | 21 (5.6%) |  |
|  | Widowed | 14 (2.0%) | 8 (2.4%) | 6 (1.6%) |  |
| Ethnicity | Han ethnicity | 512 (72.2%) | 224 (66.7%) | 288 (77.2%) | 0.002 |
|  | Ethnic minorities | 197 (27.8%) | 112 (33.3%) | 85 (22.8%) |  |
| Educational level | Below senior high school | 444 (62.6%) | 228 (67.9%) | 216 (57.9%) | 0.008 |
|  | Senior high school and above | 265 (37.4%) | 108 (32.1%) | 157 (42.1%) |  |
| Smoking history | No | 371 (52.3%) | 182 (54.2%) | 189 (50.7%) | 0.392 |
|  | Yes | 338 (47.7%) | 154 (45.8%) | 184 (49.3%) |  |
| Alcohol drinking history | No | 527 (74.3%) | 255 (75.9%) | 272 (72.9%) | 0.413 |
|  | Yes | 182 (25.7%) | 81 (24.1%) | 101 (27.1%) |  |
| Prior hypoglycemia history | No | 573 (80.8%) | 242 (72.0%) | 331 (88.7%) | < 0.001 |
|  | Yes | 136 (19.2%) | 94 (28.0%) | 42 (11.3%) |  |
| Family history of diabetes | No | 593 (83.6%) | 287 (85.4%) | 306 (82.0%) | 0.266 |
|  | Yes | 116 (16.4%) | 49 (14.6%) | 67 (18.0%) |  |
| History of hypertension | No | 573 (80.8%) | 274 (81.5%) | 299 (80.2%) | 0.709 |
|  | Yes | 136 (19.2%) | 62 (18.5%) | 74 (19.8%) |  |
| Pre-existing chronic heart disease | No | 656 (92.5%) | 310 (92.3%) | 346 (92.8%) | 0.913 |
|  | Yes | 53 (7.5%) | 26 (7.7%) | 27 (7.2%) |  |
| Diabetic ketosis | No | 433 (61.1%) | 199 (59.2%) | 234 (62.7%) | 0.379 |
|  | Yes | 276 (38.9%) | 137 (40.8%) | 139 (37.3%) |  |
| Diabetic ketoacidosis | No | 622 (87.7%) | 295 (87.8%) | 327 (87.7%) | 1.000 |
|  | Yes | 87 (12.3%) | 41 (12.2%) | 46 (12.3%) |  |
| Hyperosmolar hyperglycemic state | No | 704 (99.3%) | 334 (99.4%) | 370 (99.2%) | 1.000 |
|  | Yes | 5 (0.7%) | 2 (0.6%) | 3 (0.8%) |  |
| Diabetic retinopathy | No | 592 (83.5%) | 279 (83.0%) | 313 (83.9%) | 0.831 |
|  | Yes | 117 (16.5%) | 57 (17.0%) | 60 (16.1%) |  |
| Diabetic nephropathy | No | 638 (90.0%) | 296 (88.1%) | 342 (91.7%) | 0.143 |
|  | Yes | 71 (10.0%) | 40 (11.9%) | 31 (8.3%) |  |
| Diabetic peripheral neuropathy | No | 215 (30.3%) | 86 (25.6%) | 129 (34.6%) | 0.0118 |
|  | Yes | 494 (69.7%) | 250 (74.4%) | 244 (65.4%) |  |
| Diabetic macrovascular complications | No | 516 (72.8%) | 235 (69.9%) | 281 (75.3%) | 0.127 |
|  | Yes | 193 (27.2%) | 101 (30.1%) | 92 (24.7%) |  |
| Diabetic foot | No | 703 (99.2%) | 334 (99.4%) | 369 (98.9%) | 0.778 |
|  | Yes | 6 (0.8%) | 2 (0.6%) | 4 (1.1%) |  |
| Sleep disturbance | No | 690 (97.3%) | 327 (97.3%) | 363 (97.3%) | 1.000 |
|  | Yes | 19 (2.7%) | 9 (2.7%) | 10 (2.7%) |  |
| Polypharmacy | No | 126 (17.8%) | 65 (19.3%) | 61 (16.4%) | 0.346 |
|  | Yes | 583 (82.2%) | 271 (80.7%) | 312 (83.6%) |  |
| Use of oral antidiabetic agents | No | 358 (50.5%) | 182 (54.2%) | 176 (47.2%) | 0.075 |
|  | Yes | 351 (49.5%) | 154 (45.8%) | 197 (52.8%) |  |
| Continuous glucose monitoring application | No | 695 (98.0%) | 327 (97.3%) | 368 (98.7%) | 0.313 |
|  | Yes | 14 (2.0%) | 9 (2.7%) | 5 (1.3%) |  |
| Insulin pump therapy | No | 635 (89.6%) | 275 (81.8%) | 360 (96.5%) | < 0.001 |
|  | Yes | 74 (10.4%) | 61 (18.2%) | 13 (3.5%) |  |
| Multiple insulin formulations application | No | 217 (30.6%) | 109 (32.4%) | 108 (29.0%) | 0.355 |
|  | Yes | 492 (69.4%) | 227 (67.6%) | 265 (71.0%) |  |

Table S3 Full results of univariate logistic regression for hypoglycemia in hospitalized patients with LADA

| **Variables** | | **OR** | **95% CI** | ***p* value** |
| --- | --- | --- | --- | --- |
| Largest amplitude of glycemic excursions (mmol/L) | | 1.156 | 1.111-1.204 | < 0.001 |
| Fasting C-peptide (ng/mL) | | 0.511 | 0.398-0.655 | < 0.001 |
| 2-hour postprandial C-peptide (ng/mL) | | 0.785 | 0.717-0.860 | < 0.001 |
| Length of hospital stay (days) | | 1.140 | 1.091-1.190 | < 0.001 |
| Low-density lipoprotein cholesterol (mmol/L) | | 0.706 | 0.591-0.843 | < 0.001 |
| Body mass index (BMI) | | 0.916 | 0.871-0.964 | < 0.001 |
| Diabetes duration (years) | | 1.050 | 1.024-1.076 | 0.001 |
| Hemoglobin (g/L) | | 0.989 | 0.981-0.996 | 0.003 |
| Total cholesterol ( mmol/L) | | 0.845 | 0.747-0.957 | 0.008 |
| Estimated glomerular filtration rate (mL/min/1.73m^2^) | | 0.991 | 0.985-0.998 | 0.008 |
| Postprandial glucose excursion (mmol/L) | | 0.946 | 0.896-0.998 | 0.044 |
| Triglyceride (mmol/L) | | 0.882 | 0.778-0.999 | 0.049 |
| Serum potassium (mmol/L) | | 1.338 | 0.969-1.847 | 0.077 |
| White blood cell count (×10^9^/L) | | 0.948 | 0.894-1.006 | 0.079 |
| Glycated hemoglobin (%) | | 0.959 | 0.913-1.007 | 0.092 |
| Serum calcium (mmol/L) | | 0.505 | 0.210-1.215 | 0.128 |
| Preprandial insulin dosage (IU/d) | | 0.989 | 0.976-1.003 | 0.137 |
| Serum creatinine (μmol/L) | | 1.003 | 0.999-1.007 | 0.170 |
| Smoking duration (years) | | 0.994 | 0.983-1.004 | 0.222 |
| Age (years) | | 1.006 | 0.996-1.017 | 0.243 |
| Total daily insulin dosage (IU/d) | | 0.994 | 0.984-1.005 | 0.304 |
| Red blood cell count (×10^12^/L) | | 0.945 | 0.845-1.057 | 0.325 |
| Serum sodium (mmol/L) | | 1.019 | 0.981-1.059 | 0.333 |
| Total serum protein (g/L) | | 0.992 | 0.975-1.009 | 0.365 |
| Alcohol drinking duration (years) | | 0.995 | 0.983-1.008 | 0.429 |
| Mean in-hospital blood glucose (mmol/L) | | 1.018 | 0.957-1.083 | 0.571 |
| High-density lipoprotein cholesterol (mmol/L) | | 0.989 | 0.942-1.039 | 0.665 |
| Age at diabetes onset (years) | | 0.998 | 0.987-1.008 | 0.679 |
| Long-acting insulin dosage (IU/d) | | 1.000 | 0.979-1.021 | 0.976 |
| Insulin pump therapy | Yes | 6.143 | 3.308-11.407 | < 0.001 |
| Prior hypoglycemia history | Yes | 3.061 | 2.052-4.566 | < 0.001 |
| Sex | Female | 0.637 | 0.490-0.898 | 0.008 |
| Diabetic peripheral neuropathy | Yes | 1.537 | 1.111-2.127 | 0.010 |
| Use of oral antidiabetic agents | Yes | 0.756 | 0.562-1.016 | 0.064 |
| Diabetic macrovascular complications | Yes | 0.762 | 0.547-1.062 | 0.107 |
| Diabetic nephropathy | Yes | 1.491 | 0.910-2.444 | 0.113 |
| Continuous glucose monitoring application | Yes | 2.026 | 0.672-6.106 | 0.210 |
| Family history of diabetes | Yes | 0.780 | 0.522-1.166 | 0.225 |
| Marital status | Divorced | 1.407 | 0.776-2.550 | 0.261 |
| Polypharmacy | Yes | 0.815 | 0.554-1.198 | 0.299 |
| Diabetic ketosis | Yes | 1.159 | 0.857-1.568 | 0.339 |
| Smoking history | Yes | 0.869 | 0.647-1.168 | 0.352 |
| Alcohol drinking history | Yes | 0.855 | 0.610-1.200 | 0.366 |
| Marital status | Widowed | 1.492 | 0.512-4.346 | 0.463 |
| Diabetic foot | Yes | 0.552 | 0.100-3.034 | 0.494 |
| History of hypertension | Yes | 0.914 | 0.628-1.330 | 0.640 |
| Hyperosmolar hyperglycemic state | Yes | 0.738 | 0.122-4.443 | 0.740 |
| Diabetic retinopathy | Yes | 1.066 | 0.717-1.585 | 0.753 |
| Pre-existing chronic heart disease | Yes | 1.075 | 0.614-1.882 | 0.801 |
| Diabetic ketoacidosis | Yes | 0.988 | 0.630-1.549 | 0.958 |
| Marital status | Married | 0.995 | 0.671-1.474 | 0.979 |
| Sleep disturbance | Yes | 0.999 | 0.401-2.489 | 0.999 |
| Geographic origin | Chongqing Municipality | 0.357 | 0.259-0.493 | < 0.001 |
| Ethnicity | Ethnic minorities | 1.694 | 1.216-2.360 | 0.006 |
| Educational level | Senior high school and above | 0.652 | 0.479-0.886 | 0.013 |
| Multiple insulin formulations application | Yes | 0.849 | 0.616-1.169 | 0.327 |

Table S4 Sensitivity analysis of multivariate logistic regression across imputed datasets

| **Variables** | **OR**  **(20 imputations)** | ***p* value**  **(20 imputations)** | **OR**  **(10 imputations)** | ***p* value**  **(10 imputations)** | **Relative difference in OR (%)** | **Difference in *p* value** |
| --- | --- | --- | --- | --- | --- | --- |
| Total cholesterol (mmol/L) | 1.051 | 0.726 | 1.055 | 0.711 | -0.355 | 0.015 |
| Low-density lipoprotein cholesterol (mmol/L) | 0.777 | 0.181 | 0.776 | 0.184 | 0.092 | -0.004 |
| 2-hour postprandial C-peptide (ng/mL) | 0.945 | 0.461 | 0.930 | 0.382 | 1.553 | 0.079 |
| Fasting C-peptide (ng/mL) | 0.743 | 0.103 | 0.762 | 0.189 | -2.522 | -0.086 |
| Estimated glomerular filtration rate (mL/min/1.73m²) | 0.993 | 0.121 | 0.993 | 0.117 | 0.012 | 0.004 |
| Geographic origin | 0.369 | < 0.001 | 0.366 | < 0.001 | 0.932 | < 0.001 |
| Hemoglobin (g/L) | 0.996 | 0.546 | 0.996 | 0.509 | 0.027 | 0.037 |
| Glycated hemoglobin (%) | 0.902 | 0.007 | 0.901 | 0.007 | 0.211 | < 0.001 |
| Triglyceride (mmol/L) | 1.013 | 0.846 | 1.016 | 0.807 | -0.328 | 0.039 |
| Diabetes duration (years) | 1.029 | 0.097 | 1.030 | 0.096 | -0.029 | 0.001 |
| Sex | 1.655 | 0.025 | 1.640 | 0.028 | 0.922 | -0.003 |
| Ethnicity | 1.542 | 0.054 | 1.537 | 0.056 | 0.312 | -0.001 |
| Body mass index (kg/m²) | 1.005 | 0.872 | 1.008 | 0.828 | -0.215 | 0.045 |
| Diabetic peripheral neuropathy | 1.003 | 0.989 | 1.005 | 0.983 | -0.166 | 0.006 |
| Largest amplitude of glycemic excursions (mmol/L) | 1.136 | < 0.001 | 1.137 | < 0.001 | -0.167 | < 0.001 |
| Prior hypoglycemia history | 2.447 | < 0.001 | 2.407 | < 0.001 | 1.626 | < 0.001 |
| Length of hospital stay (days) | 1.134 | < 0.001 | 1.135 | < 0.001 | -0.118 | < 0.001 |
| Educational level | 0.838 | 0.392 | 0.841 | 0.401 | -0.279 | -0.009 |
| White blood cell count (×10^9^/L) | 0.952 | 0.182 | 0.953 | 0.195 | -0.170 | -0.013 |
| Insulin pump therapy | 8.223 | < 0.001 | 8.049 | < 0.001 | 2.120 | < 0.001 |
| Postprandial glucose excursion (mmol/L) | 0.968 | 0.378 | 0.974 | 0.471 | -0.616 | -0.093 |
| Serum potassium (mmol/L) | 1.066 | 0.720 | 1.062 | 0.728 | 0.388 | -0.008 |
| Use of oral antidiabetic agents | 0.942 | 0.755 | 0.928 | 0.700 | 1.404 | 0.055 |
