# Supplementary figures and images for: Risk of Hypoglycemia and Associated Factors Among In‐Hospital Chinese Patients With Latent Autoimmune Diabetes in Adults (LADA): A Multicenter Retrospective Cohort Study
Source: J Diabetes Res. 2026 Jun 29;2026:2961800. doi: 10.1155/jdr/2961800 (PMC13312302; doi:10.1155/jdr/2961800)

Average Correlation Matrix (Mixed Types, 20 imputed datasets)

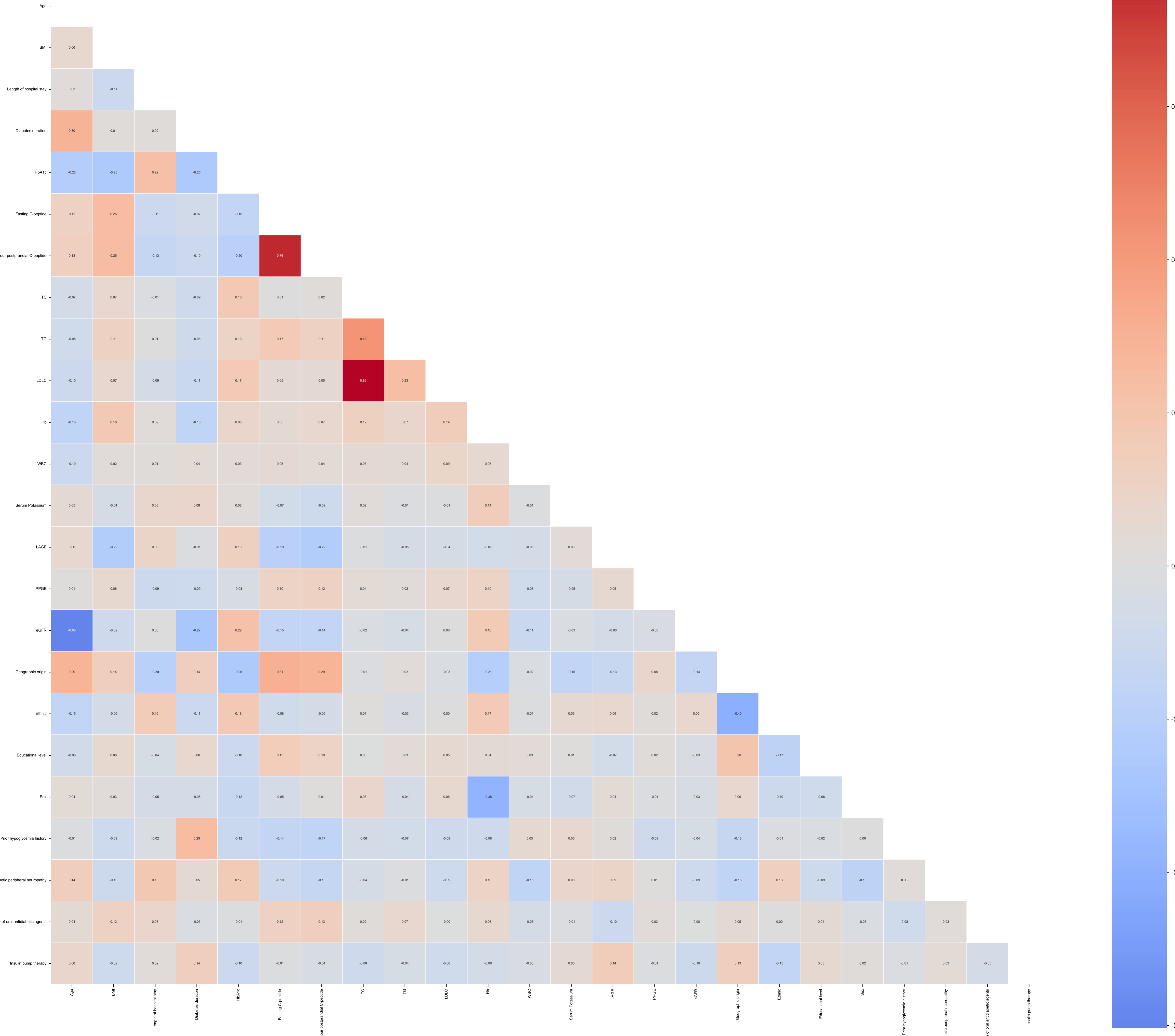

Supplement: Supplementary file 2 — Supporting Information 2 Figure S1: Heatmap of averaged correlation coefficients among screened variables derived from 20 multiply imputed datasets. [file JDR-2026-2961800-s002.pdf]
